# Targeted PLGA–Chitosan Nanoparticles for NIR-Triggered Phototherapy and Imaging of HER2-Positive Tumors

**DOI:** 10.3390/pharmaceutics16010009

**Published:** 2023-12-20

**Authors:** Polina A. Kotelnikova, Victoria O. Shipunova, Sergey M. Deyev

**Affiliations:** 1 Shemyakin-Ovchinnikov Institute of Bioorganic Chemistry, Russian Academy of Sciences, 16/10 Miklukho-Maklaya St., 117997 Moscow, Russia; 2 Moscow Institute of Physics and Technology, 9 Institutskiy Per., 141701 Dolgoprudny, Russia; 3 Nanobiomedicine Division, Sirius University of Science and Technology, 1 Olympic Ave., 354340 Sochi, Russia; 4 Bionanophotonics Laboratory, Institute of Engineering Physics for Biomedicine (PhysBio), National Research Nuclear University MEPhI (Moscow Engineering Physics Institute), Kashirskoe Shosse 31, 115409 Moscow, Russia; 5 Institute of Molecular Theranostics, Sechenov First Moscow State Medical University, 119991 Moscow, Russia

**Keywords:** PLGA, HER2, trastuzumab, photodynamic therapy, IR-780, Nile Blue, image-guided therapy, polymeric nanoparticles

## Abstract

Targeted medicine uses the distinctive features of cancer cells to find and destroy tumors. We present human epidermal growth factor receptor 2 (HER2)-targeted PLGA–chitosan nanoparticles for cancer therapy and visualization. Loading with two near-infrared (NIR) dyes provides imaging in the NIR transparency window and phototherapy triggered by 808 nm light. Nile Blue (NB) is a biocompatible solvatochromic NIR dye that serves as an imaging agent. Laser irradiation of IR-780 dye leads to a temperature rise and the generation of reactive oxygen species (ROS). Resonance energy transfer between two dyes allows visualization of tumors in a wide range of visible and IR wavelengths. The combination of two NIR dyes enables the use of nanoparticles for diagnostics only or theranostics. Modification of poly(lactic-co-glycolic acid) (PLGA)–chitosan nanoparticles with trastuzumab provides an efficient nanoparticle uptake by tumor cells and promotes more than sixfold specificity towards HER2-positive cells, leading to a synergistic anticancer effect. We demonstrate optical imaging of the HER2-positive mouse mammary tumor and tumor-specific accumulation of PLGA–IR-780–NB nanoparticles in vivo after intravenous administration. We managed to achieve almost complete suppression of the proliferative activity of cells in vitro by irradiation with an 808 nm laser with a power of 0.27 W for 1 min at a concentration at which nanoparticles are nontoxic to cells in the dark.

## 1. Introduction

Fluorescence tomography and fluorescence-guided surgery are currently becoming indispensable tools in cancer diagnosis and treatment [1]. Several fluorescent imaging agents are approved, e.g., Hexvix and Cysview (hexaminolevulinate) for blue-light cystoscopic detection of bladder carcinoma [2], and Gleolan 5-aminolevulinic acid (5-ALA) hydrochloride for the visualization of gliomas [3]. Approved in 1956, indocyanine green (ICG) remained the only FDA-approved NIR dye for a long time. Only at the end of 2021 did the FDA approve Cytalux (Pafolacianine, OTL38, folate receptor-targeting NIR fluorescent conjugate) for ovarian cancer lesion imaging [4] and in 2022 for lung lesions visualization.

Photodynamic (PDT) and photothermal therapies (PTT) provide a double selectivity affecting only irradiated cells after the accumulation of a photosensitizer. Light energy transforms the photosensitizer into an excited singlet state [5]. Photosensitizers can return to the ground state via a radiative (fluorescence) or nonradiative way (heating) or undergo intersystem crossing and transform into the excited triplet state. An extended lifetime allows molecules in the triplet states to react with other molecules nearby, which leads to the formation of free radicals or reactive oxygen species (ROS). These highly active molecules cause oxidative damage and cell death.

A number of drugs are approved for clinical use in PDT, e.g., porfimer sodium (Photofrin), aminolevulinic acid (Levulan), methyl aminolevulinate (Metvix), Verteporfin (Visudyne), meta-tetrahydroxy phenyl chlorin (Foscan), and talaporfin sodium (Laserphyrin) [6]. However, effective PDT requires not only light and a photosensitizer but also a sufficient concentration of oxygen in the cytoplasm [7,8].

Photothermal therapy (PTT) can be applied for hypoxic tumor treatment because it does not depend on the oxygen level [9,10,11]. PTT utilizes the increased sensitivity of cancer cells to temperatures rising up to 41–47 °C [12,13,14,15,16]. Local hyperthermia was approved for cancer treatment and radiosensitization [17]. However, medications and nanoagents for photothermal therapy are still only at the stage of preclinical and clinical trials. 

IR-780 iodide is a lipophilic near-infrared heptamethine fluorescent dye. Due to the fluorescence in the transparency window of biological tissues, it can be used for phototherapy and tumor imaging. According to the literature, once inside the cell, IR-780 can accumulate in mitochondria, leading to oxidative stress after irradiation [18]. The optical and sensitizing properties of IR-780 surpass ICG [19,20,21,22], e.g., ICG cannot be used for PDT as its singlet oxygen quantum yield is fairly low [23]. However, the hydrophobicity of IR-780 limits its use in clinical practice. To improve its biocompatibility, the dye can be loaded into the polymer or lipid shell. Incorporation of lipophilic dyes into a multicomponent polymer particle has an advantage over being enclosed in a hydrophobic core of core–shell nanoparticles (NP), due to the reduction in quenching at high dye concentrations. The administration method and the stabilizing shell influence the dye release rate, biodistribution, accumulation in the tumor, and even fluorescence efficiency. In other words, the dye “package” plays a key role in IR-780 effectiveness [24,25].

The diagnostic applications of nanoparticles can be expanded by using the biocompatible NIR dye, namely, Nile Blue (NB) [26]. Nile Blue is a cationic oxazine dye. Nile Blue can be efficiently accumulated in lysosomes and can potentially act as a lysosomotropic agent [27,28]. Its photokilling abilities depend on specific structural modifications. Nile Blue A, which was used in the current work, showed a very low photosensitizing ability and ^1^O_2_ yields, and can be used as a relatively biocompatible NIR imaging agent [29,30], while other Nile Blue derivatives can exhibit photokilling properties [31,32,33,34].

In this work, we loaded IR-780 and Nile Blue into PLGA–chitosan nanoparticles. We encapsulated the dye in biodegradable PLGA–chitosan nanoparticles by the double emulsion method. Thanks to its outstanding biocompatibility, PLGA was approved in the clinic for biodegradable surgical and scaffolding material design and as a drug carrier for the treatment of oncological, endocrine, infectious, and cardiac diseases. By modifying nanoparticles with additional polymers, it is possible to regulate cell binding and the rate of drug release.

To demonstrate targeted delivery, we chose trastuzumab as an antitumor agent with proven effectiveness [35,36]. In the work of Colzani et al., trastuzumab was added at the first step of the PLGA NP synthesis as a cargo. It was shown that trastuzumab could be released from the PLGA depot within 30 days without changing the protein structure. At the same time, its ability to bind to the receptor and penetrate cancer cells and its therapeutic effects were superior to free antibodies [37]. Several studies have shown not only trastuzumab loading into a polymer particle but also adsorption on the NP surface, with the formation of a rigid protein corona [38]. Specific cell binding gives us a reason to believe that trastuzumab remains available for receptor recognition within the particle. Along with the particle degradation, trastuzumab can be released gradually, providing a synergistic anticancer effect. 

## 2. Materials and Methods

### 2.1. Chemicals

IR-780 iodide (Sigma-Aldrich, Saint Louis, MO, USA), Nile Blue A perchlorate (Sigma-Aldrich, Saint Louis, MO, USA), Poly(D,L-lactide-co-glycolide) (RG 858 S, Poly(D,L-lactide-co-glycolide) ester terminated, lactide:glycolide 85:15, Mw 190,000–240,000 Da, Sigma, Darmstadt, Germany), PVA (Mowiol 4-88, Sigma, Steinheim, Germany), chitosan oligosaccharide lactate (5 kDa, Sigma, Steinheim, Germany), penicillin/streptomycin (Paneco, Moscow, Russia), 2 mM L-glutamine (Paneco, Moscow, Russia), DMEM/F12 with 2 mM L-alanyl-L-glutamine (Gibco, Paisley, UK), Hoechst33342 (Thermo Fisher Scientific, Waltham, MA, USA), Versene solution (Paneco, Moscow, Russia), penicillin-streptomycin (Paneco, Moscow, Russia), bovine serum albumin (Paneco, Moscow, Russia), Herceptin (Roche, Mannheim, Germany), Crystal violet dye (LenReaktiv, St. Petersburg, Russia), fetal bovine serum (HyClone, Logan, UT, USA), Carboxy-H_2_DCFDA, (Thermo Fisher Scientific, Waltham, MA, USA), and resazurin sodium salt (AlfaAesar, Lancashire, UK). 

### 2.2. Cell Culture

SKBR3, A549, CHO, BT474 cell lines (Shemyakin-Ovchinnikov Institute RAS, Molecular Immunology Laboratory collection) were cultured in DMEM/F12 medium supplemented with 10% FBS, 2 mM L-alanyl-L-glutamine, and penicillin/streptomycin under a humidified atmosphere at 37 °C and 5% CO_2_.

Versene solution (EDTA solution in PBS) was used to remove cells from culture plastic to prevent destruction and removal of surface receptors by trypsin.

### 2.3. Nanoparticle Synthesis 

**Figure 1:**
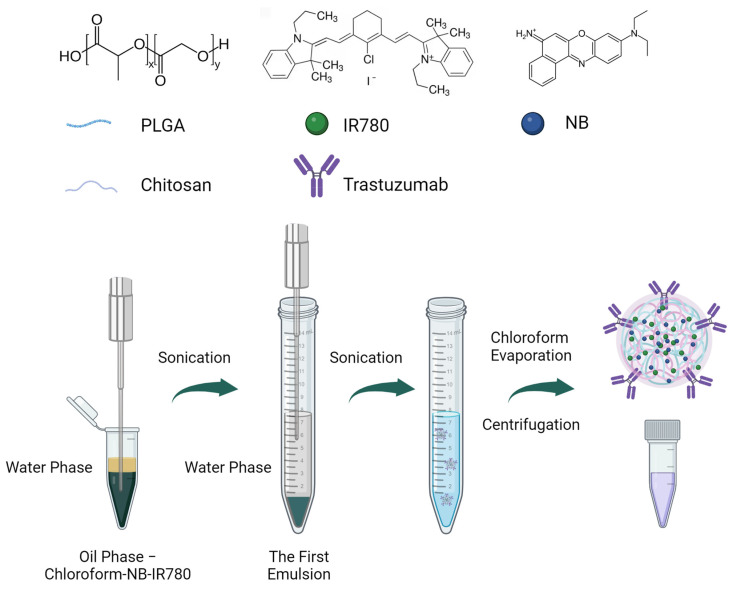
General scheme of nanoparticle synthesis. The first emulsion was obtained by sonication dye solution in chloroform with water phase. Then, this emulsion was placed in the tube with PVA and protein solution in PBS and emulsified by ultrasound. After chloroform evaporation, NPs were washed with PBS and ready to use.

Nanoparticles were prepared by the double emulsion method, developed by us earlier, with modifications [39,40,41,42] (Figure 1).

A total of 12 mg PLGA was dissolved in 300 μL chloroform, then 25 μL Nile Blue (1 g/L) and 25 μL IR-780 (3 g/L) in chloroform were added. For the first emulsion, 150 μL of water was added to PLGA and sonicated for 1 min for 1 min using an ultrasonic processor VCX750 (Sonics & Materials, Newtown, CT, USA) equipped with a stepped tip 1/8″—630-0422 with maximum power of 750 W and output frequency of 20 kHz. Samples were sonicated at 38% amplitude for 1 min. Then the first emulsion was transferred to the 15 mL centrifuge tube containing 1.125% PVA, 0.625% BSA, 0.3 mg chitosan oligosaccharide lactate, and 0.75 mg trastuzumab in 3 mL PBS and sonicated at 38% amplitude for 1 min. The solvent was evaporated under a hood while gently mixing at reciprocal mode on the Biosan RS-24 rotator for at least 4 h. Next, NPs were washed three times with PBS by centrifugation and resuspended in 200 μL of PBS. Nanoparticle concentration was determined after aqueous suspension drying at 70 °C. 

### 2.4. Nanoparticle Characterization

Nanoparticle morphology and size were studied with an MAIA3 (Tescan, Brno, Czech Republic) scanning electron microscope. Briefly, 1 µL of nanoparticles 50 µg/L in water was applied on a silicon wafer and air-dried. SEM images were obtained at an accelerating voltage of 7 kV. Next, SEM images were processed using ImageJ 1.53k software. The sizes of at least 1000 nanoparticles were measured, and then the size distribution was plotted and analyzed using GraphPad Prism 9.5.1 software.

Hydrodynamic size and ζ-potential of the nanoparticles were measured by Zetasizer Nano ZS (Malvern Instruments, Malvern, Worcestershire, UK). Measurements were performed in PBS (hydrodynamic size) or 10 mM NaCl (ζ-potential). Number size distribution was used for analysis. 

The optical properties of NP were studied using an Infinite M1000 PRO (Tecan, Salzburg, Austria) microplate reader. 

To measure the dye loading efficiency, 10 µL of NPs were dissolved in 90 µL of DMSO. Calibration solutions were prepared by dissolving dyes in DMSO since IR-780 is insoluble in water. Absorbance spectra were obtained with the Infinite M1000 PRO microplate reader. The optical density of a well with 100 µL of DMSO was used as a baseline.

To measure the fluorescence of nanoparticles in cells, cells were seeded at 15 × 10^3^ cells/well in 100 µL of a phenol-red-free cell medium. Then, 10 µL of NP (30 g/L) were added to the cells in triplets and incubated for 4 h at 37 °C to allow the particles to enter the cells. Then, the fluorescence spectra were recorded using the Infinite M1000 PRO microplate reader.

### 2.5. NP Toxicity Studies

SKBR3 cells were seeded 3 × 10^3^ cells/well in a 96-well plate and cultivated overnight at 37 °C, 5% CO_2_. The next day, 100 µL of nanoparticle suspension was added to cells and incubated for 72 h. Then, cells were carefully washed with warm PBS two times, and 100 µL of resazurin solution (13 mg/L) in a cultural medium was added. After 3–5 h of incubation at 37 °C and 5% CO_2_, the fluorescence was measured at wavelengths of λ_ex_ = 570 nm and λ_em_ = 600 nm using an Infinite M1000 PRO (Tecan, Austria) microplate reader. Nontreated cells were taken as 100%, and wells with resazurin solution were taken as a baseline.

### 2.6. Flow Cytometry Binding Assay

Cell lines A549, CHO, SKBR3, and BT474 were resuspended as 10^6^ cells/mL in phenol-red-free cell medium, 10% FBS. NPs (10 µL, 30 g/L) were added to 300 µL of cell suspension. Cells were incubated at 4 °C for 30 min under continuous slow stirring. Cells were separated from unbound NPs by centrifugation at 100 g for 5 min twice, resuspended in 100 µL DMEM/F12 (phenol-red-free) cell medium, and analyzed using Novocyte 3000 VYB flow cytometer (ACEA Biosciences, San Diego, CA, USA) with 561 nm excitation laser and 660/20 nm emission filter.

### 2.7. ROS Generation

Cells in phenol-red-free cell medium were incubated with nanoparticles at 37 °C for 1 h with gentle stirring every 15 min. Samples were irradiated for 0, 10, 30, or 60 s with 808 nm laser at a power of 1.2 W. ROS indicator (carboxy-H_2_DCFDA) was added to samples according to manufacturer recommendations. After incubation in the dark, cells were immediately analyzed using flow cytometer Novocyte 3000 VYB flow cytometer at excitation laser 488 nm, emission filter 530/30 nm. Hydrogen peroxide (to obtain a final concentration of 0.03%) was added to positive controls.

### 2.8. Study of Photothermal Properties 

To evaluate the photothermal properties of PLGA–IR780–NB nanoparticles, 100 µL of NP suspension in PBS was irradiated in 1.5 mL tubes with 808 nm laser at the power of 1.2 W and 0.27 W. Temperature measurement data were obtained using a thermal imaging camera FLIR C3 (FLIR Systems, Wilsonville, OR, USA), and the temperature of the spots with the highest temperature was used for analysis. The initial temperature of the tube stored at room temperature (RT) was used as the baseline. A tube with 100 µL of PBS was used as a control.

### 2.9. Phototherapy In Vitro

SKBR3 cells were resuspended as 10^6^ cells/mL in phenol-red-free DMEM/F12 cell medium. NPs (18 uL, 30 g/L) were added to 450 µL cell suspension in 1.5 mL centrifuge tubes. After incubation at 37 °C for 1 h with gentle stirring, every 15 min cells were separated from unbound NPs by centrifugation at 100g for 5 min and resuspended in 450 µL DMEM/F12 phenol-red-free cell medium. Then, 100 µL of the sample was placed in 1.5 mL tubes and irradiated for 0, 1.5, or 15 min with 808 nm laser at a power of 0.27 W. After irradiation, 1.4 mL of DMEM/F12 was added to each sample and mixed very carefully.

For phototoxicity assay, 100 µL of cell suspension was placed in a well of a 96-well plate and cultivated for 48 h. Next, 100 µL of resazurin solution in a cultural medium was added. After 3–5 h of incubation, the fluorescence was measured using the microplate reader.

For the clonogenic assay, 10^3^ cells after irradiation were placed in each well of a 12-well plate in 1 mL of DMEM/F12 with 10% FBS. Cells were cultivated for 10 days to form colonies with medium change every 3 days. Then, medium was removed and wells were washed sequentially with 1 mL PBS, 50%, and 75% ethanol in PBS. Next, cells were fixed with 96% ethanol for 15 min. Then ethanol was removed and the wells were washed with water. Cells were stained with 1% crystal violet water solution for 30 min. Then, the dye was removed, and the wells were washed with water. Cell plates were scanned with an Epson Perfection 2400 scanner (Epson, Batam, Indonesia).

### 2.10. Tumor Bearing Mice 

Female BALB/c mice of 22–25 g weight were purchased from Stolbovaya Breeding Center (Stolbovaya, Moscow Oblast, Russia) and maintained at the Vivarium of the IBCh RAS (Moscow, Russia). All procedures were approved by the Institutional Animal Care and Use Committee (IACUC) of the Shemyakin-Ovchinnikov Institute of Bioorganic Chemistry Russian Academy of Sciences (Moscow, Russia) according to the IACUC protocol #367/2022.

Female mice were injected with 1 × 10^6^ EMT6/P-HER2 cells [41] in 100 µL of culture media in the mammary fat pad to obtain the orthotopic tumors [43]. Before tumor inoculation, mice were anesthetized by inhalation of a 1.5% isoflurane/oxygen gas mixture using a Rodent Anesthesia System (Perkin Elmer, Hopkinton, MA, USA).

### 2.11. In Vivo Tumor Imaging 

On the eighth day after tumor inoculation, mice were intravenously injected with 1 mg of PLGA–IR-780–NB nanoparticles in 100 µL of PBS into the retroorbital sinus under gas anesthesia.

After 24 h, mice were anesthetized with 1.5% isoflurane/oxygen gas mixture for 3–5 min and transferred into IVIS Spectrum CT (Perkin Elmer, Hopkinton, MA, USA) bioimaging system. The maintenance of the anesthesia was provided with a gas manifold (1.5% isoflurane/oxygen mixture). The temperature of the mouse body was maintained by the stage heating during the measurement.

### 2.12. Confocal Microscopy

SKBR3 cells were seeded on a glass bottom cell slide and incubated under a humidified atmosphere with 5% CO_2_ at 37 °C overnight. Next, Hoechst 33342 dye was added to each well at a final concentration of 1 µg/mL. After 1 h incubation, cell media were replaced with PLGA–IR-780–NB in phenol-red-free cell medium. After 2 h, unbound NPs were removed with gentle rinse with prewarmed phenol-red-free cell medium.

Confocal microscopy was performed using an LSM 980 microscope (Zeiss, Jena, Germany) under the following conditions: excitation 405 nm, emission 408–501 nm for Hoechst 33342; excitation 543 nm, emission 552–694 nm for NB.

### 2.13. Statistical Analysis

The statistical analysis was performed using GraphPad Prism 9.5.1. Unless stated otherwise, all data are shown as mean with SD. The differences were calculated by two-way ANOVA (alpha = 0.05) with Tukey’s multiple comparisons test, with asterisks indicating *p*-values in the following way: 0.12 (ns), 0.033 (*), 0.002 (**), <0.001 (***).

## 3. Results

### 3.1. Nanoparticle Synthesis and Characterization

We synthesized PLGA nanoparticles loaded with two NIR fluorescent dyes, namely, IR-780 iodide and Nile Blue. NP concentration was 31 ± 2 g/L.

**Figure 2:**
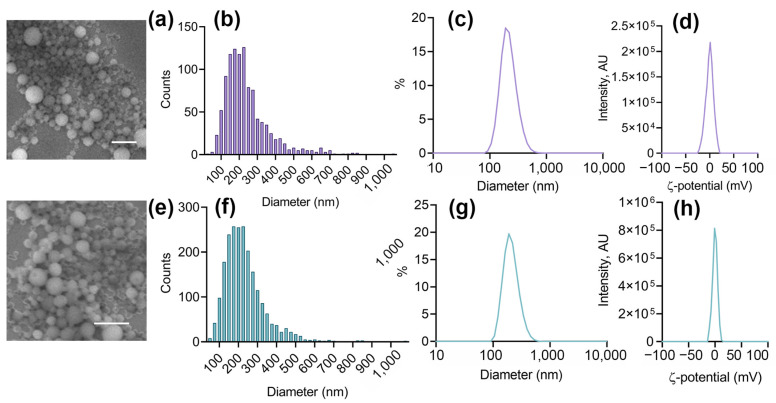
Nanoparticle characterization. (**a**,**e**) SEM images of PLGA (**a**) and PLGA–IR780–NB (**e**). Scale is 1 µM. (**b**,**f**) Size distribution of PLGA (**b**) and PLGA–IR780–NB (**f**) from SEM. (**c**,**g**) Histograms of hydrodynamic size distribution of PLGA (**c**) and PLGA–IR780–NB (**g**) nanoparticles in PBS. (**d**,**h**) Zeta-potential distributions of PLGA (**d**) and PLGA–IR780–NB (**h**) in 10 mM NaCl.

The size of the NPs was measured by DLS and SEM. According to DLS, NP sizes were 227 ± 71 nm and 222 ± 77 nm, while the size determined from SEM images was 234 ± 111 nm and 241 ± 127 nm. Zeta-potential was −0.32 ± 4.61 mV for PLGA NP and −0.41 ± 7.89 mV for PLGA–IR-780–NB NP, respectively (Figure 2).

According to absorption spectra in DMSO, IR-780 dye concentration in obtained NP was 25 µg/mL for PLGA–IR-780 NP and 23 µg/mL for PLGA–IR-780–NB NP. NB dye was 59 µg/mL PLGA–NB and 74 µg/mL for PLGA–IR-780–NB NP. The obtained data demonstrate that 50–60% of added NB and only 6–7% of IR-780 were encapsulated in PLGA NP.

**Figure 3:**
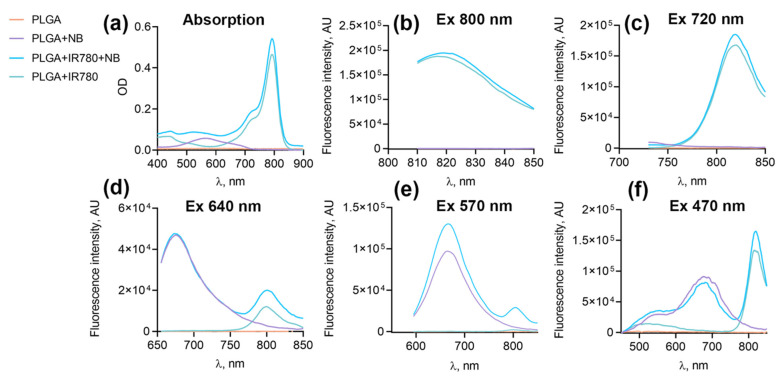
Optical properties of PLGA NPs: absorbance, fluorescence, and FRET measurements. (**a**) Absorption spectra of NP solutions. (**b**–**f**) Emission spectra of NB after excitation at 800 nm (**b**), 720 nm (**c**), 640 nm (**d**), 570 nm (**e**), and 470 nm (**f**). Additional emission peak in NIR for PLGA–IR-780–NB shows the energy transfer between two dyes.

Since the optical properties of Nile Blue are highly dependent on the solvent (solvatochromism) [26], it was important to determine whether Nile Blue fluorescence and resonance energy transfer between the two dyes would be observed after cell uptake and in tumor. Being internalized by cells, NB loaded into NP had an absorbance maximum at 570 nm and an emission maximum at 670 nm. IR-780 loaded NB had an absorbance maximum at 790 nm and an emission maximum at 820 nm (Figure 3a). IR-780 fluorescence was not excited by 570 wavelength light; however, excitation at 570 nm and 640 nm of the combination of IR-780 and NB led to two emission peaks, namely, 670 nm for NB and 820 nm for IR-780 emission (Figure 3d,e). Resonance energy transfer between dyes expands their diagnostic and therapeutic applications.

### 3.2. Study of Photothermal Properties 

**Figure 4:**
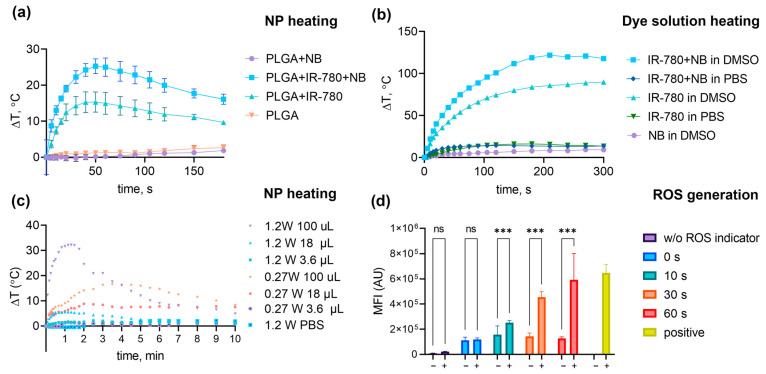
Photothermal properties and ROS generation study. (**a**) Heating of PLGA NPs loaded with loaded with NB, IR-780, or NB+IR-780 with 808 nm laser (1.2 W). (**b**) Heating-free IR-780, NB, or their combination in different solvents with 808 nm laser (1.2 W). (**c**) NP heating with 808 nm laser (1.2 W or 0.27 W). (**d**) ROS generation in HER2-positive SKBR3 cells with or without NP after NIR irradiation (808 nm, 1.2 W). Pluses correspond to samples with NPs; minuses correspond to cells without NPs. The yellow bar represents positive control (H_2_O_2_ added). The asterisks indicate *p*-values in the following way: 0.12 (ns), <0.001 (***).

PLGA–IR-780–NB NP suspension was irradiated with an 808 nm laser with a power of 1.2 W or 0.27 W. With the laser irradiation at 1.2 W, the temperature of the samples increased dramatically, reaching a maximum in 1 min, and then reduced almost to the initial value. A rapid temperature rise of more than 30 °C per minute can provide an interesting tool for tissue ablation (such as soft-tissue laser surgery, LASIK, or endovenous laser ablation). However, in most cases, gentle heating leading to apoptosis is required to avoid unwanted inflammation. Turndown of the laser power led to a smoother rise in temperature with a longer plateau (Figure 4c). Thus, we used an 808 nm laser with a power of 0.27 W for in vitro phototherapy experiments. A total of 18 µL NP (0.45 µg IR-780) in 100 µL PBS showed heating with laser irradiation (808 nm, 0.27 W) at 8.8 °C for at least 10 min without temperature diminution. Such dye concentrations can be achieved in a mouse tumor, which makes the particles promising for in vivo applications. It is worth noting that the particles loaded with Nile Blue only practically did not heat up; however, the combination of dyes resulted in more efficient heating than NP with IR-780 alone (Figure 4a). The same effect was observed for free dyes (30 mg/L) heated in DMSO. The IR-780+NB combination showed superior heating compared to pristine IR-780 at the same concentration, whereas pure NB did not heat up at all. Freshly obtained IR-780+NB and IR-780 solutions in PBS did not heat up either. This may indicate dye aggregation in PBS and confirms the need for IR-780 encapsulation in NP (Figure 4b).

### 3.3. Study of the Photosensitizing Properties of Nanoparticles

A total of 18 µL (30 g/L) NP was added to 450 µL cell suspension (final IR-780 concentration was 1.2 µM). Samples were irradiated for 10–60 s with 808 nm, 1.2 W laser. It was shown that the addition of nanoparticles to cells did not lead to a rise in the ROS level, whereas the ROS level increased in proportion to the irradiation time in cells with PLGA–IR–NB nanoparticles, but not in cells without particles (Figure 4d). 

### 3.4. PLGA NP Interaction with Cells

Four cell lines with different HER2 expression levels were selected: HER2-overexpressing human breast cancer cell lines BT474 and SKBR3, human lung carcinoma A549 with low HER2 overexpression, and Chinese hamster ovary CHO cells as HER2-negative cells.

**Figure 5:**
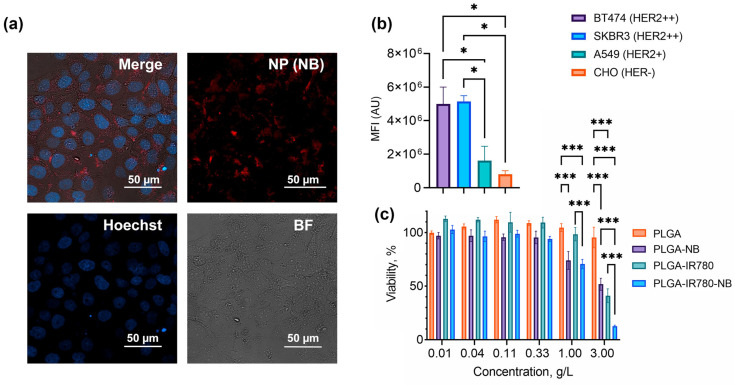
HER2-positive cell targeting with PLGA-based nanophotosensitizers: HER2-targeting efficiency assays, cytotoxicity tests. (**a**) Confocal microscopy of SKBR3 cells after incubation with PLGA+IR-780+NB nanoparticles. (**b**) NP binding with cells with different HER2 levels measured by flow cytometry. (**c**) Cytotoxicity test of NP loaded with NB, IR-780, and NB+IR-780 performed by resazurin assay. Untreated cells were used as 100%. The asterisks indicate *p*-values in the following way: 0.033 (*), <0.001 (***).

Cells were incubated with the nanoparticles, washed from nonbound nanoparticles, and analyzed using the flow cytometer in the FL channel corresponding to the fluorescence of Nile Blue. The nanoparticles showed specific binding to HER2-positive cells, namely, more than six times stronger with BT474 and SKBR3 than with HER2-negative CHO (Figure 5b). 

### 3.5. Targeted Delivery of Dye-Loaded PLGA Nanoparticles to HER2-Positive Cells: Specificity Assays and Cytotoxicity Tests 

To evaluate the toxicity of nanoparticles loaded with dyes, we added nanoparticles to the cells and incubated them without washing for 72 h (Figure 5c). The particles did not have a significant toxic effect in a wide range of concentrations. IC50 for PLGA–NB and PLGA–IR-780 were 3 g/L and 1.5 g/L for PLGA–IR-780–NB. In terms of dye concentration, the IC50 was 2.5 mg/L for IR-780 (4 mM) and 6 mg/L for NB (14 mM).

### 3.6. NIR-Light Induced Phototherapy In Vitro

**Figure 6:**
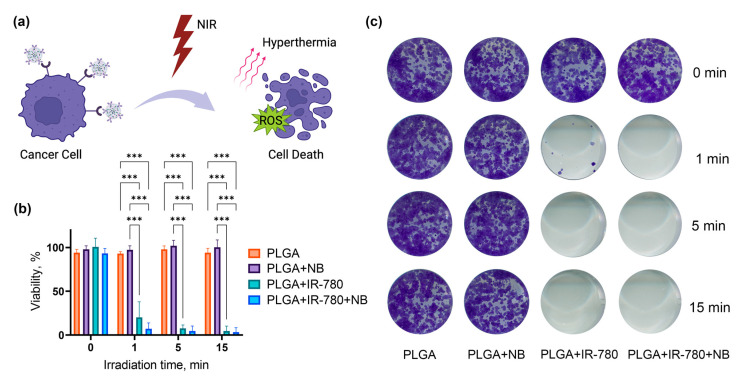
Phototherapy using PLGA–IR-780–NB NPs. (**a**) Targeted NPs bind predominantly to cancer cells. NIR irradiation of IR-780 leads to local hyperthermia and ROS generation. (**b**) Resazurin assay for SKBR3 cells irradiated with 808 nm (0.27 W) laser after incubation with PLGA, loaded with NB and/or IR-780. (**c**) Clonogenic assay for SKBR3 cells irradiated with 808 nm (0.27 W) laser after incubation with PLGA, loaded with NB and/or IR-780. The asterisks indicate *p*-values in the following way: <0.001 (***).

To evaluate the contribution of each of the dyes to photosensitization, we synthesized nanoparticles loaded with Nile Blue, IR-780, or two dyes simultaneously. Then, 18 µL of particles were added to 450 µL cell suspension (final IR-780 concentration was 1.2 µM, NB concentration was 7 mM). After incubation, the excess nanoparticles were removed by centrifugation, and then the cells were irradiated with an 808 nm laser at 0.27 W. The cells were resuspended in a complete medium and seeded into a 96-well plate. The resazurin test was used to evaluate cell viability (Figure 6b). Neither pristine PLGA NPs nor PLGA–NB NPs showed any significant phototoxicity. IR-780 and IR-780–NB NPs killed almost all cancer cells even after 1 min of NIR irradiation.

Since colorimetric tests did not show a significant difference between IR-780 and IR-780–NB effectiveness, we used a clonogenic assay. It helps to evaluate the proliferative activity of cells; in other words, the ability to form colonies from single cells. The clonogenic assay showed that after 5 and 15 min of irradiation with an 808 nm laser (0.27 W), there was not a single cell left capable of forming a colony in samples with PLGA–IR-780 and PLGA–IR-780–NB. After 1 min of irradiation, only a few colonies were formed per well from 1000 seeded cells with PLGA–IR-780 (Figure 6c). Irradiation of cells with NPs loaded with the combination of dyes led to complete cell death. It is important to note that irradiation of cells without nanoparticles, even for 15 min, did not cause a decrease in proliferative activity. 

### 3.7. Imaging Properties of PLGA–IR-780–NB Nanoparticles

**Figure 7:**
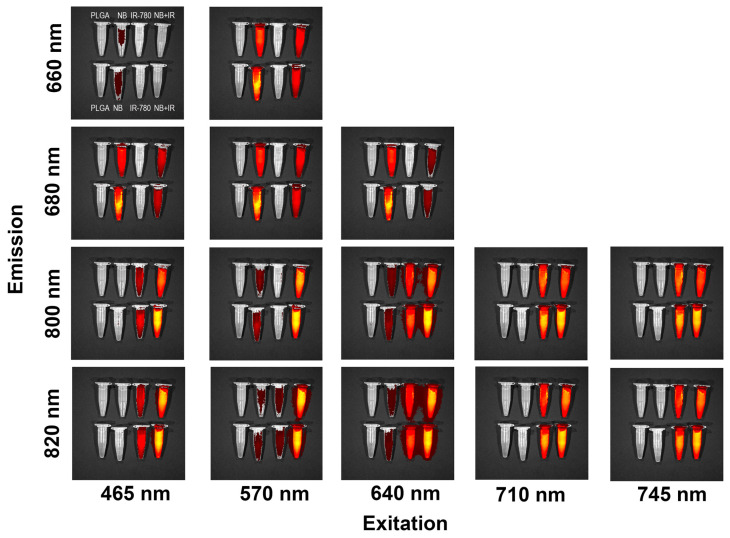
Fluorescence images of PLGA NP suspensions in PBS at 1 g/L (by IVIS optical imager). From left to right: PLGA NPs without dye, NPs loaded with Nile Blue, NPs loaded with IR-780, and NPs loaded with a combination of dyes. The top and bottom rows correspond to independently synthesized nanoparticles.

We imaged tubes with 1 g/L nanoparticles in the IVIS visualizer (Figure 7). Since both dyes have a weak absorption peak in the blue light region, we studied the excitation of fluorescence by light at 465 nm (Figure 7, the first column). At the emission maximum of Nile Blue (660–680 nm), the fluorescence of particles loaded only containing NB was observed (Figure 7, lines 1 and 2). At wavelengths of 800–820 nm, fluorescence was observed only in samples containing IR-780 (Figure 7, lines 3 and 4). When using a combination of dyes, the fluorescence at 660–680 nm faded, and at the 800–820 nm peak it enhanced. This may indicate an energy transfer from NB to IR-780. 

The 570 nm light excited predominantly Nile Blue fluorescence at 660–680 nm (Figure 7, the second column). It is worth noting that imaging at 800–820 nm was most efficient for the dye combination. At the same time, IR-780 itself was almost not excited, and did not have a photokilling effect. It is possible to visualize tumor cells in the Nile Blue channel without excitation of IR-780 phototoxic properties. Nile Blue can act as a visualization agent when excited by light with a wavelength of less than 640 nm, while IR-780 is required for visualization when excited in the red and infrared regions.

### 3.8. In Vivo Tumor Imaging

In vivo imaging was performed on the orthotopic mouse model of breast cancer [43]. Orthotopic tumor models may be more representative than subcutaneous injection in the mouse flank, as they can reproduce the tumor microenvironment more accurately [44,45]. EMT6/p is a highly tumorigenic mouse breast cancer line. EMT6/p-HER2 modification enables targeted therapy investigation on nonimmunodeficient mice [41].

Mice received an injection of 1 × 10^6^ EMT6/p-HER2 cells into the mammary gland in 100 µL of cell medium. In a week, grafted tumor had an appearance of the ellipsoidal lump approximately 5 mm in diameter in the mammary gland corresponding to the cell injection.

Mice were injected with 1 mg of PLGA–IR-780–NB NPs in PBS intravenously, and the accumulation in the tumor was studied after 24 h using an IVIS optical imager.

**Figure 8:**
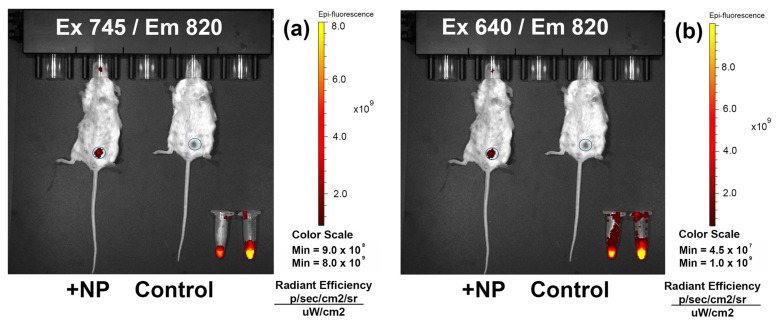
In vivo NIR visualization of HER2-positive tumor 24 h after intravenous injection obtained using IVIS optical imager with the following optical filters: (**a**) excitation 745 nm/emission 820 nm; (**b**) excitation 640 nm/emission 820 nm. Tubes contain 1 mg PLGA–IR-780–NB NPs in PBS (left) and DMSO (right).

A number of images were obtained with excitation filters 640–745 nm and emission filters 800–840 nm. Fluorescence in the area of the tumor significantly exceeded the background (Figure 8). Hair removal was not required for a well-defined tumor visualization. It is worth mentioning that after intravenous NP injection, no acute adverse reactions were observed.

PLGA–IR-780–BN NPs may be considered promising agents for tumor visualization.

## 4. Discussion

The development of targeted therapy and tumor imaging methods is essential in the fight against cancer. Traditional therapies suffer from a lack of selectivity and a considerable number of side effects. Late tumor detection is the key obstacle to effective cancer treatment and the reason for the high mortality.

The overwhelming majority of publications in the field of nanomedicine are based on the enhanced permeability and retention (EPR) effect. However, the EPR is not as effective in the human body as in rodents; it is often limited to the peritumoral area and can only be applied to some rapidly growing tumors. In the case of human tumors, the EPR is not sufficient, and targeted delivery methods are required [46,47,48].

Overexpression of the HER2 receptor is associated with an aggressive disease course and a poor prognosis for the patient [49,50]. The challenge is not only to detect a tumor but also to determine the oncomarker expression to select the correct treatment tactics. Biopsy does not always give accurate results due to the tumor heterogeneity. Therefore, specific labeling and imaging of the tumors inside the body become more important.

In this work, we synthesized HER2-targeted PLGA–chitosan nanoparticles loaded with two NIR dyes, namely, IR-780 and NB. The resulting particle size of about 200 nm still allows penetration into the tumor by means of EPR. However, trastuzumab on the surface of the obtained particles provided six times stronger binding to HER2-positive cells in vitro. We presume that trastuzumab allowed the effective accumulation and retention of nanoparticles in mouse tumors. 

NIR and visible light optical diagnostic and sensitization methods are generally safer for tissues than X-ray irradiation. However, they are associated with certain restrictions, such as limited light permeability of biological tissues. Visible light can only penetrate a few millimeters. The NIR region, the so-called biological transparency window, is considered the most beneficial for diagnostics. We obtained clear images of small breast tumors with IVIS optical imager multiple using fluorescence filters (Ex 640-745/Em 800–840 nm), even though the excitation maximum of IR-780 is between 780–800 nm, which expands the range of possible imaging systems that can be used. In previous research, IR-780-loaded particles targeted folic acid receptor [21,51], CD44 [52,53,54], or mitochondria [18,55]. We applied the monoclonal antibody trastuzumab to obtain specific binding with HER2-overexpressing cells. IR-780 showed outstanding photosensitizing abilities. Due to the synergy of PTT and PDT, one minute of irradiation was enough to suppress the proliferative activity of cells. A radically different behavior of the dye was noted at different irradiation modes. The high power resulted in rapid heating in 1 min for all three NP concentrations, followed by cooling down to baseline even with continuous irradiation. At a low laser power, a temperature plateau sufficient for successful hyperthermia persisted for at least 10 min. PTT with the use of PLGA–IR-780–NB nanoparticles can be performed under milder irradiation conditions compared to gold–silicon nanoparticles undergoing clinical trials where sub-ablative laser power and gram quantities of NPs are required [56,57]. 

The temperature drop upon irradiation with a laser with a power of 1.2 W is probably due to the photodegradation of the IR-780. The destruction of the IR-780 under laser irradiation was described in some previous studies. Encapsulation of IR-780 in a polymer, protein, or lipid shell improves photostability and phototherapy efficiency [58,59,60]. Locally, the temperature of the particles can rise significantly higher than that measured by the camera. It can lead to faster release of the dye and its degradation under irradiation. The stability and drug release time can be optimized by changing the polymer composition of the particle. The ratio between glycoside and lactate affects the melting point of the copolymer. In a number of works, PLGA nano- and microcapsules have been used for thermally activated drug release: namely, by heating magnetic nanoparticles in a magnetic field [61,62], plasmonic particles [63] under the action of light. The “melting” property of PLGA–chitosan nanoparticles can be not only a limitation but also can open two directions for further research, specifically for the development of a flash release [64] of a thermostable drug in 1 min under powerful laser exposure or long-term mild tumor hyperthermia.

## 5. Conclusions

We developed targeted PLGA–chitosan nanoparticles loaded with two NIR dyes. We hope that the synergy of PTT and PDT will make it possible to destroy tumors, including resistant and hypoxic ones. We assume that the combination of a biocompatible NP matrix, a safe tracking agent, and an excellent photosensitizer with targeted delivery may be the key to success in oncotheranostics.

## Data Availability

All the data are presented within this article. The raw datasets are available on request from the corresponding authors.
